# Glucagon-Like Peptide-1 Receptor Agonists in Inflammatory Bowel Disease: A Narrative Review

**DOI:** 10.1016/j.gastha.2026.101023

**Published:** 2026-05-28

**Authors:** Aakash Desai, Hany Habib, Lina Wahbeh, Francis A. Farraye

**Affiliations:** 1 Division of Gastroenterology, Hepatology and Nutrition, Allegheny Health Network, Pittsburgh, Pennsylvania; 2 Department of Medicine, Drexel University College of Medicine, Philadelphia, Pennsylvania; 3 Department of Medicine, Medicine Institute, Allegheny Health Network, Pittsburgh, Pennsylvania; 4 Division of Gastroenterology and Hepatology, Mayo Clinic, Jacksonville, Florida

**Keywords:** Glucagon-Like Peptide-1 Receptor Agonists, Inflammatory Bowel Disease, Obesity, Type 2 Diabetes Mellitus, Narrative Review

## Abstract

Glucagon-like peptide-1 receptor agonists (GLP-1RAs) have progressed from the initial discovery of endogenous GLP-1 biology in 1986 to widely used therapies for type 2 diabetes and obesity, with expanding indications driven by pleiotropic metabolic and cardiovascular benefits. Although no randomized controlled trials have evaluated GLP-1RA as inflammatory bowel disease (IBD)-directed therapy, retrospective observational studies in IBD populations prescribed GLP-1RA for metabolic indications provide emerging real-world evidence regarding weight loss efficacy, IBD-related outcomes, and adverse events. This narrative review summarizes GLP-1RA pharmacology, mechanistic rationale, and the current clinical evidence base in IBD, with emphasis on safety considerations and priorities for prospective trials.

## Glucagon-Like Peptide-1 Background

The identification of glucagon-like peptide-1 (GLP-1) receptor agonists (RAs) represents a landmark discovery in metabolic medicine that has evolved from basic molecular biology to transformative clinical applications. In 1986, GLP-1 was discovered through molecular cloning of complementary DNAs, revealing 2 molecular amide forms: GLP-1 (7–37) and GLP-1 (7–36).^1^ GLP-1 is primarily secreted from enteroendocrine L-cells located in the intestinal epithelium of the distal small intestine and colon, serving as a key incretin hormone in glucose homeostasis.^2^, ^3^, ^4^, ^5^ Beyond the intestinal tract, GLP-1 is also secreted by alpha cells in the pancreas, expanding our understanding of its endocrine functions.^6^ More recent investigations have demonstrated that preproglucagon-expressing neurons in the solitary tract nucleus also secrete GLP-1, potentiating central regulation of satiety and autonomic function, thereby establishing a crucial brain-gut axis mechanism.^7^ GLP-1-secreting cells have been identified in the gastric corpus/fundus region, though their contribution to circulating GLP-1 levels appears minimal compared to intestinal L-cells.^8^ The discovery that GLP-1 possessed incretin-like hormonal activity, leading to increased glucose-dependent insulin secretion while simultaneously suppressing glucagon secretion, provided the pharmacological rationale for developing GLP-1RA as therapeutic agents for diabetes mellitus.^9^

## Mechanism of Action

**Figure 1:**
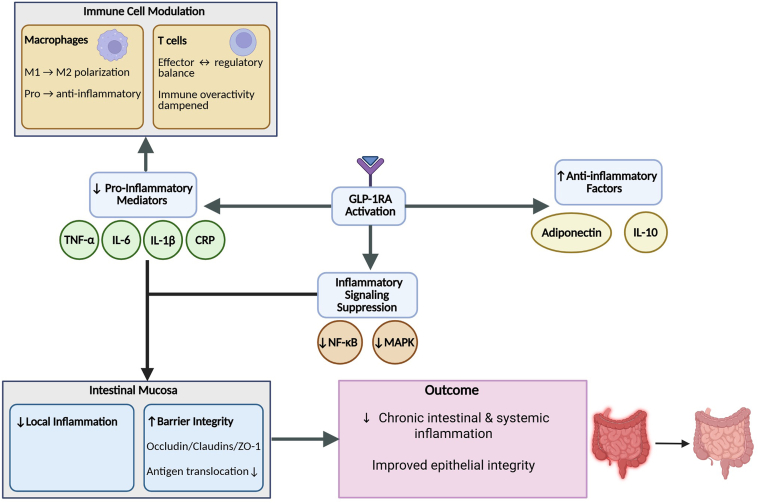
Mechanisms of action of GLP-1 receptor agonists in inflammatory bowel disease.

GLP-1RA function by mimicking the endogenous GLP-1 hormone produced by the body, though with enhanced stability and prolonged duration of action compared to native GLP-1, which is rapidly degraded by dipeptidyl peptidase-4 (DPP-4).^5^^,^^10^^,^^11^ Early clinical trials evaluating first-generation GLP-1RA, including exenatide and liraglutide, demonstrated significant reductions in both fasting and postprandial glucose levels, with the additional unexpected benefit of substantial weight loss in treated patients.^12^, ^13^, ^14^, ^15^, ^16^ The widespread distribution of GLP-1 receptors throughout the human body accounts for the pleiotropic effects of these medications. GLP-1 receptors are predominantly expressed in pancreatic beta-cells, where they potentiate insulinotropic effects and enhance glucose-dependent insulin secretion.^17^, ^18^, ^19^ These receptors are also expressed, albeit weaker, in pancreatic acinar cells.^20^ Within the gastrointestinal tract, GLP-1 receptors are highly expressed in Brunner glands of the duodenum, with lower expression levels observed in gastric parietal cells, smooth muscle cells of the stomach, and myenteric plexus neurons, contributing to delayed gastric emptying and enhanced satiety signals.^21^, ^22^, ^23^ In the central nervous system, GLP-1 receptors have been identified in multiple regions critical for metabolic regulation and appetite control, including the hypothalamus, area postrema, nucleus tractus solitarius, amygdala, and mesolimbic reward system, as well as in vagal afferent nerves that relay gut-brain signals.^24^, ^25^, ^26^, ^27^, ^28^, ^29^ Additionally, GLP-1 receptors are found in myocytes of the sinoatrial node, kidneys, lungs, blood vessels, adipose tissue, and skeletal muscle, which collectively explain the broad metabolic, cardiovascular, and systemic effects observed with GLP-1RA therapy (Figure 1).

## Development of Glucagon-Like Peptide-1 Receptor Agonists and Dual Agonists

The development of GLP-1RA has progressed from short-acting formulations to long-acting agents and dual incretin RAs (Table 1). Exenatide twice-daily (Byetta) became the first GLP-1RA approved by the US Food and Drug Administration in 2005 for the treatment of type 2 diabetes mellitus.^30^ As a short-acting compound with an elimination half-life of approximately 2.4 h, exenatide requires twice-daily subcutaneous injection, which limited patient convenience.^31^ Liraglutide (Victoza for diabetes, Saxenda for obesity) administered once daily was approved in 2010 for type 2 diabetes, followed by approval for adult obesity in 2014 and adolescent obesity (age ≥12 years) in 2020.^32^^,^^33^ Dulaglutide (Trulicity), approved in 2014 for type 2 diabetes, was developed as a long-acting compound by coupling modified GLP-1 to a large immunoglobulin fragment crystallizable, which substantially prolongs its half-life to approximately 5 days, permitting once weekly subcutaneous administration.^34^ Higher doses of dulaglutide (3 mg and 4.5 mg) have been explored in clinical trials to achieve greater weight loss efficacy beyond glycemic control.^35^ Semaglutide, available in both subcutaneous (Ozempic for diabetes, Wegovy for obesity) and oral formulations, has become one of the most widely prescribed GLP-1RA. Subcutaneous semaglutide administered once weekly subcutaneously was approved in 2017 for type 2 diabetes, in 2021 for adult obesity at a 2.4 mg weekly dose, in 2022 for adolescent obesity, and in 2024 for cardiovascular disease risk reduction in adults with obesity or overweight and established cardiovascular disease.^33^^,^^36^ The development of semaglutide for obesity was extensively studied through the landmark Semaglutide Treatment Effect in People with Obesity clinical trial program, which demonstrated unprecedented weight loss efficacy.^37^ Oral semaglutide (Rybelsus), administered once a day, approved in September 2019 for type 2 diabetes, represents the first GLP-1 RA available for oral administration.^38^^,^^39^ Tirzepatide (Mounjaro for diabetes, Zepbound for obesity), approved in 2022 for type 2 diabetes and in 2023 for adult obesity, is the first dual glucose-dependent insulinotropic polypeptide and GLP-1RA to reach clinical practice.^40^ This innovative unimolecular dual agonist was rationally designed to harness the synergistic metabolic effects of both incretin hormones, with glucose-dependent insulinotropic polypeptide acting complementarily with GLP-1 to promote greater weight loss and glycemic control than either hormone alone.^41^ In the landmark SURPASS-2 (A Study of Tirzepatide versus Semaglutide once weekly as add-on therapy to Metformin in Participants with Type 2 Diabetes) trial, tirzepatide achieved superior weight reduction and greater hemoglobin A1c (HbA1c) lowering compared to subcutaneous semaglutide 1 mg weekly, establishing a new benchmark for efficacy in obesity pharmacotherapy.^42^ The efficacy of tirzepatide for obesity was rigorously evaluated in the SURMOUNT (Study of tirzepatide for obesity related morbidity and mortality adults) clinical trial program, which demonstrated exceptional weight loss outcomes.^43^ Importantly, the pivotal phase 3 clinical trial programs that established the efficacy and safety of GLP-1RA and dual agonists enrolled patients with obesity or type 2 diabetes mellitus, while excluding those with a history of pancreatitis, clinically significant gastric emptying abnormalities, and other gastrointestinal conditions, and did not include patients with inflammatory bowel disease. The exclusion of this population from prospective trial data represents a significant knowledge gap, particularly given that GLP-1RA-associated gastrointestinal adverse events may be difficult to distinguish from symptoms of active inflammatory bowel disease (IBD).^44^ This limitation reinforces the importance of evaluating the available real-world evidence in patients with IBD, as summarized in the present review.

**Table 1:** Summary of FDA-Approved GLP-1 Receptor Agonists by Name, Indication, Route of Administration, Dosing Frequency, Half-Life, and Approval Year

Generic name	Brand name	Indication	Route	Dosing frequency	Half-life	FDA approval year
Exenatide	Byetta	T2DM	SubQ	Twice daily	2–3 h	2005
	Bydureon ER	T2DM	SubQ	Once weekly	∼6 d	**2012**
Liraglutide	Victoza	T2DM	SubQ	Once daily	13–15 h	2010
	Saxenda	Obesity	SubQ	Once daily	13–15 h	2014
Lixisenatide	Adlyxin	T2DM	SubQ	Once daily	2–4 h	**2016**
Albiglutide^a^	Tanzeum	T2DM	SubQ	Once weekly	6–8 d	2014
Dulaglutide	Trulicity	T2DM	SubQ	Once weekly	∼5 d	2014
Semaglutide	Ozempic	T2DM	SubQ	Once weekly	∼7 d	2017
	Wegovy	Obesity	SubQ/PO	Once weekly	∼7 d	2021/2025
	Rybelsus	T2DM	PO	Once daily	∼7 d	2019
Tirzepatide	Mounjaro	T2DM	SubQ	Once weekly	∼5 d	2022
	Zepbound	Obesity	SubQ	Once weekly	∼5 d	2023

Bolded entries indicate medication discontinued.

ER, extended release; PO, per os; subQ, subcutaneous; T2DM, type 2 diabetes mellitus.

a Discontinued 2018 (commercial reasons).

## Glucagon-Like Peptide-1 Effects Beyond the Pancreas

The therapeutic potential of GLP-1 RAs extends far beyond glycemic control, with emerging evidence demonstrating beneficial effects across multiple organ systems.^45^, ^46^, ^47^, ^48^, ^49^ In hepatic tissue, GLP-1RA have emerged as promising therapeutic agents for metabolic dysfunction–associated steatotic liver disease and metabolic dysfunction–associated steatohepatitis. Clinical trials have documented significant improvements in hepatic steatosis, inflammation, and fibrosis markers, with recent phase 3 data demonstrating semaglutide achievement of metabolic dysfunction–associated steatohepatitis resolution in 62.9% of patients with moderate-to-advanced fibrosis compared to 34.3% with placebo at 72 weeks.^50^, ^51^, ^52^ These pleiotropic effects underscore the broad therapeutic potential of GLP-1RA beyond their initial indication for diabetes management.

## Anti-Inflammatory Effects

GLP-1RA has demonstrated notable and clinically relevant anti-inflammatory effects through multiple molecular mechanisms that modulate both innate and adaptive immunity. These agents have been shown to suppress nuclear factor kappa B signaling, a master regulator of inflammatory gene expression, thereby attenuating downstream inflammatory cascades.^53^^,^^54^ GLP-1 receptor activation significantly reduces the production of key pro-inflammatory cytokines, including tumor necrosis factor alpha (TNF-α), interleukin-6, interleukin-1 beta, and C-reactive protein, all of which are central mediators of systemic and tissue-specific inflammation.^53^^,^^55^ Conversely, GLP-1RA increase the production of anti-inflammatory factors, including adiponectin, an adipokine with insulin-sensitizing and anti-inflammatory properties, and interleukin-10, a critical immunoregulatory cytokine that suppresses excessive immune responses.^56^ Furthermore, these agents influence macrophage polarization, promoting a shift from pro-inflammatory M1 macrophages to anti-inflammatory M2 macrophages, thereby creating a more favorable tissue microenvironment.^57^ GLP-1RA also modulate T-cell function, affecting the balance between effector and regulatory T-cell populations, which may have important implications for autoimmune and inflammatory conditions.^58^^,^^59^ Preclinical studies utilizing experimental colitis and IBD models have provided evidence that GLP-1RA exert direct anti-inflammatory effects within the intestinal mucosa.^60^ In these models, GLP-1RA treatment has been shown to reduce intestinal inflammation through downregulation of critical inflammatory signaling pathways, including nuclear factor kappa B and mitogen-activated protein kinase pathways, leading to significant reductions in the production of TNF-α, interleukin-6, and other pro-inflammatory mediators that drive mucosal injury and perpetuate chronic intestinal inflammation.^61^ GLP-1RA have demonstrated the ability to decrease mucosal injury scores and improve epithelial integrity in experimental colitis models, suggesting a protective effect on the intestinal barrier.^61^^,^^62^ These agents promote the preservation of tight junction proteins, including occludin, claudins, and zonula occludens-1, which are critical structural components that maintain the selective permeability of the intestinal epithelium.^61^ By strengthening gut barrier function, GLP-1RA reduces the translocation of luminal antigens, including bacterial products and dietary antigens that can traverse a compromised epithelium and drive persistent inflammatory responses.^60^^,^^62^

## Glucagon-Like Peptide-1 Receptor Agonists in Inflammatory Bowel Disease

There have been no randomized controlled trials specifically designed to evaluate the efficacy of GLP-1RA for the treatment of IBD. The current evidence base is derived from retrospective observational studies that have examined clinical outcomes in patients with IBD who were prescribed GLP-1RA for concurrent metabolic indications, primarily type 2 diabetes mellitus or obesity. The body of evidence has evolved since 2021, beginning with Villumsen et al^63^ who conducted a nationwide population-based cohort study using Danish registries spanning 2007–2019, employing a new-user active comparator design in 3751 patients with IBD and type 2 diabetes (982 GLP-1/DPP-4 inhibitor users; 2769 on other antidiabetics), examining the impact of these agents on a composite outcome of steroid need, TNF-α inhibitor initiation, IBD hospitalization, or surgery. In 2024, Desai et al^64^ published a retrospective cohort study using the TriNetX US database (2010–2022) with 1:1 propensity score matching, analyzing 2270 total patients on GLP-1 RAs (1130 with ulcerative colitis; 1140 with Crohn's disease) matched to control cohorts, examining 3-year risk of hospitalization requiring intravenous (IV) steroids and IBD-related surgery. Single-center studies also emerged in 2024: Ramos Belinchón et al^65^ reported on 16 obese IBD patients from a tertiary hospital in Madrid, Spain (2019–2021), evaluating the effectiveness of semaglutide or liraglutide for weight loss and impact on IBD activity scores, while St-Pierre et al^66^ conducted an observational cohort study at the University of Chicago Medicine (2021–2024) on 36 nondiabetic IBD patients, assessing the efficacy of semaglutide or tirzepatide for weight loss and changes in metabolic risk factors. Pham et al^67^ performed a case-control study at a single academic center comparing 36 patients with IBD and obesity receiving antiobesity medications, including liraglutide and semaglutide, to 36 matched non-IBD controls, evaluating percent total body weight loss and IBD flares over 12 months.

Nielsen et al^68^ conducted a nationwide Danish cohort study of 61,927 patients with IBD (4430 exposed to GLP-1 RAs; 57,497 unexposed) examining the risk of ileus and intestinal obstruction. In 2025, several large-scale studies were published. Anderson et al^69^ performed a retrospective study at a tertiary care center (2014–2024) of 120 patients with IBD, examining safety outcomes including adverse events, weight change, and clinical/endoscopic response. Clarke et al^70^ reported a retrospective cohort study within the Massachusetts General Brigham health-care network (2020–2023) of 272 IBD patients with obesity (175 completed ≥12 months of therapy), assessing tolerability and effectiveness defined as ≥5% total weight loss at 12 months. Desai et al^71^ conducted an obesity-focused retrospective cohort study using TriNetX (2021–2023), comparing 150 obese patients with IBD on semaglutide to matched non-IBD and IBD control cohorts, evaluating mean total body weight change between 6 and 15 months and risk of IBD-specific adverse events. Gorelik et al^72^ utilized the Israeli Epi-IIRN (Epidemiology group of the Israeli Inflammatory Bowel Disease Nucleus) database to conduct a nationwide cohort study of 3737 patients with IBD and type 2 diabetes (633 treated with GLP-1 analogs), employing Cox proportional hazard models with time-varying covariates to assess a composite outcome of steroid dependence, therapy escalation, hospitalization, surgery, or death. Levine et al^73^ performed a before-and-after observational study at NYU Langone Health on 224 IBD patients matched to non-IBD controls, comparing 1-year preprescription and postprescription periods to evaluate IBD exacerbation (hospitalization, steroids, escalation, or surgery) and changes in metabolic risk factors. Sehgal et al^74^ reported on 244 patients (142 Crohn's disease; 102 ulcerative colitis) at the University of Pennsylvania Health System (2017–2023), examining the safety profile including adherence and adverse events, weight loss efficacy, and impact on inflammatory biomarkers. Thin and Teh^62^ published a systematic review evaluating 20 IBD-related studies (9 preclinical and 11 human clinical), examining the translational evidence for GLP-1RA in IBD with emphasis on whether these agents could induce steroid-free clinical remission and additionally reviewed the evidence for GLP-1RA use in non-IBD immune-mediated inflammatory diseases. Bayoumy et al^75^ published a systematic review and meta-analysis, aggregating data from the above-mentioned 11 observational studies encompassing 16,242 patients to examine weight loss and IBD clinical outcomes including hospitalization, surgery, corticosteroid use, and advanced therapy initiation. Aksan et al^76^ conducted a retrospective propensity score-matched cohort study using the TriNetX Analytics Research Network (2006–2022) encompassing 11,016 matched patients (5508 in the GLP-1 RA group; 5508 in the control group), examining 5-year mortality, IBD-related surgeries, complications, and health-care resource utilization. Weng et al^77^ reported a retrospective cohort study of 271 adult patients with IBD within an academic medical system, comparing intervals 1 year preceding and following GLP-1 RA exposure to assess the incidence of gastrointestinal adverse events including ileus, surgery, hospitalization, and therapy escalation. Desai et al^78^ conducted a retrospective cohort study using TriNetX (2022–2023) in 274 patients with IBD initiated on tirzepatide, comparing mean total body weight change from baseline to between 6 and 15 months to propensity-matched non-IBD controls on tirzepatide and to 185 patients with IBD who underwent weight loss surgery, and assessing the 1-year risk of IBD-related complications compared to matched IBD controls not on tirzepatide or other antiobesity medications. Maracle et al^44^ conducted the most recent systematic review to date, evaluating 14 clinical studies across weight-related, metabolic, IBD-specific, and safety outcome domains, and concluded that GLP-1RA appear well tolerated in patients with IBD with no evidence of increased disease exacerbation. These retrospective analyses and systematic reviews have provided safety data and information regarding potential disease-modifying effects, including impacts on hospitalization rates, surgical interventions, disease flares, corticosteroid requirements, and the need for advanced therapies such as biologics or small molecule drugs. A summary of these are presented in Table 2.

**Table 2:** Summary of GLP-1 Receptor Agonist Studies in Patients With Inflammatory Bowel Disease

Study (author and year)	Country	GLP-1 used	Patients (n)	UC/CD/both	Clinical outcomes	Main results
Villumsen et al (2021)^63^	Denmark	GLP-1RAs and DPP-4 inhibitors	3751 total (982 users)	Both (UC and CD)	Need for steroids, TNFi, hospitalization, or surgery	52% lower risk of adverse clinical events compared to other antidiabetics
Ramos Belinchón et al (2024)^65^	Spain	Semaglutide or liraglutide	16	Both (9 CD, 7 UC)	Weight change, safety, IBD activity scores	Median 6.2% weight reduction; favorable safety profile; no significant change in IBD activity
Desai et al (2024)^64^	United States	Liraglutide, dulaglutide, semaglutide	2270 (1130 UC, 1140 CD)	Both (UC and CD)	Hospitalization (IV steroids), IBD-related surgery	Significantly lower risk of surgery in both UC and CD; no difference in steroid use
Desai et al (2025; online 2024)^71^	United States	Semaglutide	150	Both (66% CD)	Total body weight change and IBD outcomes	Effective weight loss (>5%); no increased risk of IBD-specific adverse events
Levine et al (2025; online 2024)^73^	United States	Multiple (semaglutide predominant)	224	Both (43% UC, 45% CD)	IBD exacerbation and metabolic risk	No change in exacerbation rates; weight loss comparable to non-IBD populations
Gorelik et al (2025; online 2024)^72^	Israel	GLP-1 analogs	3737 total (633 treated)	Both (50.4% UC)	Composite: Steroids, escalation, hospitalization, surgery, death	Reduced risk of poor outcomes (aHR 0.74), most pronounced in obese patients
St-Pierre et al (2024)^66^	United States	Semaglutide or tirzepatide	36	Both (24 CD, 12 UC)	BMI and weight change, safety, metabolic risk	Significant BMI reduction; manageable adverse effects; no flare induction
Pham et al (2024)^67^	United States	Liraglutide, semaglutide (and other AOMs)	36 IBD	Both (21 CD, 15 UC)	% total body weight loss at 12 mo, adverse events, IBD flares	Similar weight loss at 12 mo (6.9% IBD vs 8.1% control, *P* = .30); 19.4% experienced IBD flares, all managed medically
Anderson et al (2025; online 2024)^69^	United States	Multiple (semaglutide predominant)	120	Both (61 CD, 59 UC)	Adverse events, weight loss, CRP, clinical/endoscopic response	Significant CRP reduction; safe weight loss; no change in hospitalizations
Nielsen et al (2025; online 2024)^68^	Denmark	Semaglutide, liraglutide	61,927 (4430 exposed)	Both (UC and CD)	Ileus or intestinal obstruction	No increased risk of ileus or obstruction in IBD patients
Sehgal et al (2025)^74^	United States	Multiple (semaglutide predominant)	244	Both (142 CD, 102 UC)	Adherence, adverse events, inflammatory biomarkers	Approximately 5% weight loss; significant CRP reduction; high adherence
Clarke et al (2025)^70^	United States	Semaglutide, dulaglutide, liraglutide	272	Both (137 CD, 130 UC)	≥5% weight loss and IBD flares	61% achieved ≥5% weight loss; no difference in flare rates
Thin and Teh (2025)^62^	International (SR)	Various	20 IBD studies (9 preclinical, 11 human clinical)	Both (UC and CD)	Preclinical anti-inflammatory mechanisms, composite outcomes, weight loss, safety	Preclinical anti-inflammatory and barrier-protective effects; reduced surgery, hospitalization, and steroid use; weight loss comparable to non-IBD populations
Bayoumy et al (2025)^75^	International (SR and meta-analysis)	Various	16,242 across 11 studies	Both (UC and CD)	Hospitalization, surgery, advanced therapy	Lower risk of surgery and hospitalization, particularly in obese patients
Weng et al (2025)^77^	United States	Semaglutide, dulaglutide, tirzepatide	271	Both (174 UC, 97 CD)	Ileus, surgery, hospitalization, therapy escalation	No increase in GI adverse events over 12 mo; supports clinical safety
Aksan et al (2025)^76^	United States	Multiple	11,016 total (5508 users)	Both (CD and UC)	Mortality, surgeries, complications	Lower 5-y mortality; reduced risk of surgeries and complications
Desai et al (2025)^71^	United States	Tirzepatide	274	Both (47.8% CD, 52.2% UC)	TBW change, IBD complications	Effective weight loss (>10%), comparable to non-IBD; lower than weight loss surgery; lower any-cause hospitalization (aOR 0.48)
Maracle et al (2026)^44^	International (SR)	Various	14 clinical studies (13 cohort, 1 case-control)	Both (UC and CD)	Weight, metabolic markers, IBD activity, hospitalization, surgery, safety	Significant weight loss in 10/14 studies; no increased IBD exacerbations; reduced hospitalization and surgery risk; safety consistent with non-IBD populations

AOM, anti-obesity medications; CRP, C-reactive protein; GI, gastrointestinal; TNFi, tumor necrosis factor inhibitor.

## Glycemic Control and Hemoglobin A1c Outcomes

The impact of GLP-1RA on glycemic control in patients with IBD, as measured by HbA1c, demonstrates consistent improvements in patients with concurrent type 2 diabetes mellitus, while showing minimal to no change in nondiabetic populations (Table 3). Levine et al reported a significant decrease in median HbA1c in the overall IBD cohort from 6.5% to 6.2% over 12 months (*P* = .01). When stratified by diabetes status, this improvement was driven primarily by the diabetic subgroup, which showed a significant reduction from median 7.0% (interquartile range [IQR] 6.3%–8.1%) to 6.6% (IQR 6.1%–7.6%) (*P* = .02), while the nondiabetic subgroup demonstrated a nonsignificant decrease from median 5.4% (IQR 5.0%–5.8%) to 5.3% (IQR 5.0%–5.5%) (*P* = .54).^73^ Belinchón et al similarly observed modest reductions in a predominantly obese IBD cohort, with median HbA1c decreasing from 6.2% (IQR 5.5%–7.8%) to 5.9% (IQR 5.3%–7.58%), though this did not reach statistical significance (*P* = .14). Among the 4 diabetic patients in this cohort, glycated hemoglobin decreased by 0.3% after 6 months of treatment.^65^ St-Pierre et al, focusing exclusively on nondiabetic IBD patients treated with semaglutide or tirzepatide, reported a numerical decrease in median HbA1c from 5.45% (IQR 5.3%–5.7%) to 5.3% (IQR 5.0%–5.6%), representing a reduction of 0.2%; however, this did not achieve statistical significance (*P* = .0536).^66^ In comparative effectiveness studies, Desai et al (2024) found that GLP-1RA achieved similar glycemic control to other oral hypoglycemic agents in propensity score-matched cohorts. In the ulcerative colitis cohort with type 2 diabetes, mean HbA1c during follow-up was nearly identical between the GLP-1 RA group (7.18%) and the control group (7.17%) (*P* = .83). Similarly, in the Crohn's disease cohort, mean HbA1c was 7.2% in the GLP-1 RA group compared to 7.1% in controls (*P* = .05). A subgroup analysis examining semaglutide specifically showed mean HbA1c of 6.8% compared to 7.0% in controls, though this difference was not statistically significant (*P* = .12).^64^ In a separate study evaluating tirzepatide specifically, Desai et al^78^ reported a significant reduction in HbA1c among patients with IBD, with a mean change of −0.76 (95% confidence interval [CI] −0.99 to −0.52), which was slightly less than the −0.88 reduction observed in propensity-matched non-IBD controls (*P* < .0001). The systematic review by Bayoumi et al (2025)^75^ acknowledged that glycemic control improved significantly in several individual studies but did not perform a meta-analysis for HbA1c due to heterogeneity, in which specific GLP-1RA was responsible for the observed decreases and differences in baseline patient characteristics. Maracle et al^44^ similarly noted that 4 of the included studies demonstrated improvements in HbA1c, though 2 of these did not reach statistical significance. Overall, the evidence indicates that GLP-1RA provide effective glycemic control in patients with IBD with concurrent diabetes mellitus, achieving HbA1c reductions comparable to or potentially superior to other antidiabetic medications, while producing minimal changes in HbA1c levels in nondiabetic patients with IBD.

**Table 3:** Metabolic Outcomes With GLP-1 Receptor Agonist in Patients With IBD

Study (year)	Weight/BMI outcomes	HbA1c change
Ramos Belinchón et al (2024)^65^	Median TBW loss 6.2% (92.17→81.95 kg); 58.3% achieved ≥5% TBW loss; 16.7% achieved ≥10% TBW loss	6.2 → 5.9; −0.3% (NS; *P* = .14); diabetic subgroup: −0.3%
Desai et al (2024)^64^	Mean weight 214.2 lbs (UC) and 217.5 lbs (CD); mean BMI 34.4 (UC) and 34.6 (CD)	7.18 (UC); 7.20 (CD) → comparable to controls; no difference vs controls (UC: NS, *P* = .83; CD: borderline, *P* = .05)
St-Pierre et al (2024)^66^	Median weight loss 8.15 kg; BMI 34.0→31.0 kg/m^2^; continuing therapy achieved median TBW loss 11.5%	5.45 → 5.30; −0.15% (NS; *P* = .053)
Pham et al (2024)^67^	Mean %TBWL 6.9% ± 8.3% at 12 mo; comparable to non-IBD controls (8.1% ± 7.0%; *P* = .30)	—
Anderson et al (2025)^69^	Mean TBW loss 3.9%; semaglutide subgroup 5.3% TBW loss	—
Sehgal et al (2025)^74^	Mean weight 102→97.6 kg (≈5% TBW loss); semaglutide subgroup 6.2% TBW loss	—
Desai et al (2025)^71^	Mean weight loss 16 ± 13.4 lbs; TBW loss 7% at 6–12 mo and 9% at 12–15 mo	—
Thin and Teh (2025)^62^	Weight loss in IBD patients comparable to non-IBD populations. Preclinical animal models also showed slower body weight loss in liraglutide-treated mice compared to controls	—
Bayoumy et al (2025) (meta-analysis)^75^	Pooled weight loss: semaglutide −9.1 to −9.6 kg; liraglutide −9.0 to −9.4 kg; tirzepatide −11.6 to −11.8 kg	Variable across studies; directionally favorable; not pooled
Clarke et al (2025)^70^	Mean TBW loss 8.72% ± 8.98%; BMI reduction −3.26 ± 3.60 kg/m^2^; 61% achieved ≥5% TBW loss; 42% achieved ≥10% TBW loss	—
Levine et al (2025)^73^	Median BMI 33.5→31.6 kg/m^2^; clinically significant loss in patients with baseline BMI ≥30 kg/m^2^	6.5 → 6.2; −0.3% (*P* = .01); diabetic subgroup: −0.4% *P* = .02)
Weng et al (2025)^77^	Median BMI 33.8→33.4 kg/m^2^; change not statistically significant (*P* = .20)	—
Desai et al (2025)^71^	Mean TBW loss −21 lbs (>10%); comparable to non-IBD on tirzepatide (−21 lbs, *P* = 1.0); lower than weight loss surgery (−55 lbs, *P* < .0001)	−0.76 (95% CI −0.99 to −0.52); non-IBD: −0.88 (95% CI −1.13 to −0.62; *P* < .0001)
Maracle et al (2026)^44^	10/14 studies reported significant reductions in body weight, BMI, or percent weight loss	

BMI, body mass index; CD, Crohn's disease; NS, not significant; TBW, total body weight; UC, ulcerative colitis.

## Weight Change

GLP-1RA demonstrates consistent and clinically significant weight loss in patients with IBD, with outcomes comparable to non-IBD populations (Table 3). The meta-analysis by Bayoumi et al quantified agent-specific weight loss, finding that tirzepatide produced the greatest median weight loss at −11.6 kg (95% CI: −18.3 to −4.8, *P* = .0008), followed by semaglutide at −9.1 kg (95% CI: −11.8 to −6.4, *P* < .00001) and liraglutide at −9.0 kg (95% CI: −12.7 to −5.3, *P* < .00001), while dulaglutide showed no statistically significant weight loss (median −3.9 kg, 95% CI: −9.7–1.9, *P* = .19).^75^ Maracle et al^44^ reported in their recent systematic review that 10 of 14 included studies demonstrated significant reductions in body weight, body mass index (BMI), or percent weight loss, and Thin and Teh (2025)^62^ similarly concluded that weight loss in IBD patients treated with GLP-1RA was comparable to non-IBD populations across the evaluated studies. Desai et al reported that semaglutide resulted in a mean total body weight change of approximately −16 ± 13.4 pounds (about −7.26 kg) in obese patients with IBD, with no significant difference compared to non-IBD controls (*P* = .24), demonstrating that IBD status does not impair weight loss efficacy. Agent comparisons showed tirzepatide achieved greater loss than semaglutide (−26 lbs vs −18 lbs, *P* = .01), and semaglutide achieved greater loss than liraglutide (−19 lbs vs −13 lbs, *P* = .04). In a separate study evaluating tirzepatide, Desai et al reported a mean weight loss of −21 lbs (>10%) in patients with IBD, which was identical to propensity-matched non-IBD controls (*P* = 1.0). A dose-response effect was observed, with 15 mg tirzepatide achieving −29 lbs compared to −15 lbs with 5 to 10 mg doses. However, tirzepatide-associated weight loss remained significantly lower than that achieved with weight loss surgery (−55 lbs, *P* < .0001).^78^ Clarke et al found a mean total weight loss of 8.72 ± 8.98% at 12 months in a large real-world cohort, with mean BMI change of −3.26 ± 3.60 kg/m^2^. In this study, 61% of patients achieved at least 5% weight loss and 42% achieved 10% or greater weight loss. Semaglutide demonstrated the highest efficacy, with 76.0% of patients achieving 5% weight loss (*P* = .02), compared to only 42.31% with dulaglutide.^71^ Multiple smaller studies corroborated these findings, including St-Pierre et al^66^ showing median weight loss of 8.15 kg (*P* < .0001) with BMI decreasing from 34.0 to 31.0 kg/m^2^ (*P* < .0001), Levine et al^73^ demonstrated significant BMI reduction from 33.5 to 31.6 kg/m^2^ (*P* < .01), and Sehgal et al^74^ reported mean weight loss of 5.2 ± 5.0% (from 102 kg to 97.6 kg, *P* < .01), with semaglutide achieving 6.2% loss. Belinchón et al^65^ found median percentage change of −6.2% at 6 months (*P* = .002), with weight decreasing from 92.17 kg to 81.95 kg. Anderson et al confirmed that semaglutide achieved the most significant weight reduction at −5.3% compared to an overall mean of −3.9%. Notably, weight loss efficacy was observed in both diabetic (BMI 33.2–32.6 kg/m^2^, *P* = .04) and nondiabetic patients with IBD (BMI 33.5–31.5 kg/m^2^, *P* < .01), supporting the use of these medications for weight management across the IBD population spectrum.^69^ Pham et al^67^ reported comparable weight loss between IBD and non-IBD patients on antiobesity medications, with the IBD cohort achieving a mean %TBWL of 6.9% ± 8.3% at 12 months compared to 8.1% ± 7.0% in matched non-IBD controls (*P* = .30). One study by Weng et al^77^ found only modest, nonsignificant BMI changes from 33.8 to 33.4 kg/m^2^ (*P* = .20), which may have been limited by lack of dosage information. Sehgal et al^74^ found that patients with ulcerative colitis had significant weight loss of 4.73 kg (*P* = .01), while patients with Crohn's disease did not show statistical significance in multivariable regression. The evidence overwhelmingly supports that GLP-1RA are effective weight loss medications in patients with IBD comparable to the general population.

## Surgical Intervention

The evidence regarding surgical intervention risk in patients with IBD using GLP-1RA shows mixed but generally favorable results across multiple studies (Table 4). Desai et al found that overall GLP-1RA use was associated with a significantly lower risk of IBD-related surgery in patients with CD compared to oral hypoglycemic agents, with an adjusted hazard ratio (aHR) of 0.55 (95% CI: 0.36–0.84), representing a reduction in surgical intervention from 5.4% in controls to 3.2% in the GLP-1 RA group. When examining specific agents, semaglutide demonstrated the strongest protective effect with an aHR of 0.27 (95% CI: 0.13–0.57), while dulaglutide (aHR: 1.01, 95% CI: 0.55–1.85) and liraglutide (aHR: 0.80, 95% CI: 0.32–1.98) showed no significant difference. In head-to-head comparisons with other antidiabetic medication classes in CD, GLP-1 RA demonstrated significantly lower surgical risk compared to DPP-4 inhibitors (aHR: 0.37, 95% CI: 0.15–0.89), SGLT2 inhibitors (aHR: 0.36, 95% CI: 0.14–0.89), and sulfonylureas (aHR: 0.41, 95% CI: 0.20–0.80). Similarly, in patients with UC, GLP-1RA use was associated with a significantly reduced risk of total colectomy with an aHR of 0.37 (95% CI: 0.14–0.97) compared to other oral hypoglycemic agents. Class-specific comparisons in ulcerative colitis demonstrated significant reductions in colectomy risk compared to sulfonylureas (aHR: 0.08, 95% CI: 0.01–0.67) and metformin (aHR: 0.17, 95% CI: 0.03–0.77), though no significant difference was observed when compared specifically to DPP-4 inhibitors or SGLT2 inhibitors.^64^ Aksan et al (2025) corroborated these findings, reporting that GLP-1RA exposure was associated with a statistically lower rate of IBD-related surgeries in both Crohn's disease (hazard ratio [HR]: 0.45, 95% CI: 0.30–0.70, *P* = .001) and UC (HR: 0.50, 95% CI: 0.32–0.77, *P* = .007) over 5 years.^76^ However, Gorelik et al^72^ found no significant association between GLP-1RA use and IBD-related surgery risk in either CD (aHR: 0.94, 95% CI: 0.47–1.91) or UC (aHR: 0.74, 95% CI: 0.27–2.07) when analyzed as individual outcomes, though they did observe reduced composite poor outcome risk in obese subgroups with CD (aHR: 0.60, 95% CI: 0.45–0.79) and UC (aHR: 0.63, 95% CI: 0.48–0.89). The meta-analysis by Bayoumi et al^75^ found no significant reduction in surgical risk for either CD (log HR: 0.68, 95% CI: 0.37–1.26, *P* = .22) or UC (log HR: 0.79, 95% CI: 0.43–1.45, *P* = .44). Villumsen et al (2021)^63^ reported a numerically lower but not statistically significant risk of surgery (IRR: 0.79, 95% CI: 0.57–1.09). Pham et al^67^ reported that none of the 36 IBD patients receiving antiobesity medications, including liraglutide and semaglutide, required IBD-related surgery during the 12-month follow-up period. Similarly, Desai et al^78^ reported that fewer than 10 patients required IBD-related surgery in both the tirzepatide and matched IBD control cohorts, precluding formal statistical comparison. The systematic reviews by Thin and Teh^44^ and Maracle et al^62^ both corroborated these findings, noting that large population-based registries generally reported reduced risks of IBD-related surgery among GLP-1RA users, though neither identified prospective data to confirm a causal relationship. These conflicting results suggest that while some observational studies indicate a potential protective effect, particularly with semaglutide, the overall evidence remains inconclusive and may be influenced by patient selection factors and obesity status.

**Table 4:** Disease Control Outcomes With GLP-1 Receptor Agonist Use in IBD

Study (year)	Surgical outcomes	Advanced therapy escalation	Hospitalization outcomes
Villumsen et al (2021)^63^	IBD-related major surgery: aIRR 0.79 (NS; *P* < .05)	TNF-α inhibitor initiation: Adjusted IRR 0.56 (NS; *P* < .05)	IBD-related hospitalization: Adjusted IRR 0.73 (*P* < .05)
Ramos Belinchón et al (2024)^65^	IBD-related surgery: No surgeries observed	Biologic therapy intensification: No escalation observed	General hospital admission: No hospitalizations observed
Desai et al (2024)^64^	Colectomy (UC): aHR 0.37 (*P* < .05); IBD-related surgery (CD): aHR 0.55 (*P* < .05)	Advanced therapy initiation (bio-naïve): UC aHR 0.82 (NS); CD aHR 1.31 (NS)	Hospitalization for IV steroids: UC aHR 1.21 (NS); CD aHR 1.04 (NS)
Pham et al (2024)^67^	IBD-related surgery: No surgeries observed	Change in biologic therapy: 5 of 7 flaring patients (13.9% of total cohort)	IBD-related hospitalization: 2 of 36 patients
Gorelik et al (2025)^72^	IBD-related surgery: aHR 0.84 (NS); composite outcome: HR 0.74 (*P* < .05)	—	IBD-related hospitalization: Adjusted HR 0.74 (95% CI 0.61–0.91; *P* < .05)
Desai et al (2025)^71^	IBD-related surgery: 0% vs5.9% in controls	—	Any-cause hospitalization: aOR 0.35 (95% CI 0.19–0.67; *P* < .05)
Bayoumy et al (2025)^75^	IBD-related surgery: OR 0.46; logHR 0.61 (*P* < .05)	Advanced therapy initiation: No association with GLP-1 RA use (NS)	IBD-related hospitalization: Reduced risk in patients with BMI ≥30 (logHR 0.79; *P* < .05)
Levine et al (2025)^73^	IBD-related surgery: 1.3% preinitiation vs 1.8% postinitiation (NS; *P* = 1.00)	Medication escalation or change: 16.1% preinitiation vs 12.5% postinitiation (NS; *P* = .34)	IBD-related hospitalization: 8.9% preinitiation vs 7.6% postinitiation (NS)
Anderson et al (2025)^69^	—	—	IBD-related hospitalization: 10% preinitiation vs 10% postinitiation (NS)
Aksan et al (2025)^76^	IBD-related surgery (5-y): 2.3% vs 3.4%; HR 0.53 (*P* < .05)	Advanced therapy initiations: Median 4 vs 6 in controls (*P* < .0001)	Inpatient encounters: Lower utilization in GLP-1 RA cohort (*P* < .05)
Weng et al (2025)^77^	Bowel surgery: 3.8% preinitiation vs 2.5% postinitiation (NS; *P* = .617)	Advanced therapy escalation: 12.5% preinitiation vs 10.0% postinitiation (NS; *P* = .617)	IBD-primary diagnosis hospitalization: 2.5% preinitiation vs 0% postinitiation (NS)
Clarke et al (2025)^70^	—	Therapy escalation (flare composite): No difference in flare-related events (NS; *P* = .40)	IBD-related ED or hospital visit (flare composite): 17% preinitiation vs 13% postinitiation (NS)
Desai et al (2025)^71^	IBD-related surgery: <10 patients in both tirzepatide and control cohorts	New advanced therapy initiation: 3.5% vs 4.7%; aOR 0.73 (NS; *P* = .43)	Any-cause hospitalization: 12.2% vs 22.4%; aOR 0.48 (*P* < .0001)

aHR, adjusted hazard ratio; aIRR, adjusted incidence rate ratio; aOR, adjusted odds ratio; CD, Crohn's disease; CI, confidence interval; ED, emergency department; IRR, incidence rate ratio; IV, intravenous; NS, not significant; OR, odds ratio; TNF, tumor necrosis factor; UC, ulcerative colitis.

## Advanced Therapy Initiation and Escalation

The impact of GLP-1RA on the need for advanced IBD therapies, including biologics and small molecule drugs, has been extensively studied with generally neutral findings (Table 4). The meta-analysis by Bayoumi et al (2025)^75^ found no overall effect of GLP-1RA use on advanced therapy initiation in patients with IBD (log HR: 0.96, 95% CI: 0.74–1.23, *P* = .72), with no significant differences observed in either UC (log HR: 0.78, 95% CI: 0.53–1.14, *P* = .20) or CD (log HR: 1.10, 95% CI: 0.77–1.55, *P* = .61) subgroups. Desai et al^64^ similarly found no statistically significant difference in first-time advanced therapy initiation within 3 years for either patients with UC (aHR: 0.82, 95% CI: 0.51–1.33) or CD (aHR: 1.31, 95% CI: 0.86–2.00) using GLP-1RAs compared to controls. Gorelik et al^72^ reported no significant association between GLP-1RA use and treatment escalation in IBD (aHR: 0.82, 95% CI: 0.59–1.17), UC (aHR: 0.70, 95% CI: 0.37–1.33), or CD (aHR: 0.92, 95% CI: 0.61–1.39). However, Aksan et al presented contrasting data, finding a statistically significant lower median number of advanced therapeutic initiation instances in GLP-1 RA-exposed patients over 5 years (median 4 vs 6 events, *P* < .0001, effect size r = −0.11), with UC showing a moderate effect (median 4 vs 7 events, *P* < .0001, r = −0.17) and CD showing a negligible effect (median 5 vs 7 events, *P* < .0001, r = −0.03).^76^ Several studies, including Levine et al^73^ (16.1% pre vs 12.5% post, *P* = .34) and Weng et al^77^ (12.5% pre vs 10.0% post, *P* = .617), found no significant differences in therapy escalation rates. Villumsen et al^63^ observed a nonsignificant trend toward decreased need for TNF-α inhibitor initiation (aIRR: 0.56, 95% CI: 0.32–1.00). Desai et al^71^ found no difference in bio-naïve patients (adjusted odds ratio [aOR]: 1.03, 95% CI: 0.41–2.60, *P* = .94). Desai et al^78^ reported no significant difference in new advanced therapy initiation between the tirzepatide cohort (3.5%) and matched IBD controls (4.7%) (aOR 0.73, 95% CI 0.33–1.59, *P* = .43). Pham et al observed that 5 of 7 patients (19.4%) who experienced IBD flares while on antiobesity medications required a change in biologic therapy, though the authors noted that this rate was consistent with expected annual flare rates in the general IBD population.^67^ The systematic reviews by Thin and Teh^44^ and Maracle et al^62^ similarly noted that GLP-1RA use was not associated with increased therapy escalation, with smaller clinical cohorts showing stable rates and larger registries suggesting potential protective trends. Overall, the preponderance of evidence suggests that GLP-1RA use does not significantly increase the need for treatment escalation in patients with IBD, though a modest protective effect cannot be definitively ruled out.

## Corticosteroid Initiation

The relationship between GLP-1RA use and corticosteroid requirements in patients with IBD shows nuanced patterns across different studies and patient populations (Table 4). Villumsen et al^63^ found that GLP-1RA use was associated with a significantly decreased risk of oral corticosteroid treatment in the overall IBD cohort, with an adjusted incidence rate ratio of 0.54 (95% CI: 0.41–0.70) compared to other antidiabetics. Gorelik et al^72^ similarly reported a significantly reduced risk of steroid dependency overall (aHR: 0.66, 95% CI: 0.48–0.99), though subgroup analyses by disease type did not reach statistical significance for UC (aHR: 0.75, 95% CI: 0.42–1.35) or CD (aHR: 0.66, 95% CI: 0.41–1.08). The meta-analysis by Bayoumi et al found no overall effect on corticosteroid initiation (log HR: 1.02, 95% CI: 0.86–1.22, *P* = .79), with no significant differences in UC (log HR: 1.02, *P* = .91) or CD (log HR: 0.90, *P* = .68) subgroups. However, a sensitivity analysis limited to patients with BMI less than 30 identified a significantly lower risk (log HR: 0.71, 95% CI: 0.57–0.88, *P* = .002), suggesting obesity status may modify the relationship.^75^ Desai et al^64^ found no statistically significant differences in either oral steroid use for UC (aHR: 1.12, 95% CI: 0.96–1.31) or CD (aHR: 1.10, 95% CI: 0.95–1.28), or IV steroid use for UC (aHR: 1.21, 95% CI: 0.92–1.59) or CD (aHR: 1.04, 95% CI: 0.80–1.34). In another study, Desai et al^71^ also did not find any significant difference in corticosteroid prescription for both oral (aOR: 0.81, 95% CI: 0.48–1.36) and IV steroids (aOR: 0.69, 95% CI: 0.29–1.61). In a tirzepatide-focused study, Desai et al reported a numerically lower rate of oral steroid use in the tirzepatide group (18.5%) compared to matched IBD controls (23.4%), though this did not reach statistical significance (aOR 0.74, 95% CI 0.54–1.02, *P* = .07). No significant difference was observed in IV steroid use between the tirzepatide (17.6%) and control (15.2%) cohorts (aOR 1.19, 95% CI 0.83–1.69, *P* = .32).^78^ Cohort studies consistently reported no significant changes in corticosteroid prescription rates following GLP-1RA initiation, including Levine et al^73^ (22.3% pre vs 18.3% post, *P* = .27), Weng et al^77^ (20.0% pre vs 17.5% post, *P* = .831). Pham et al^67^ reported that 4 of 36 IBD patients (11.1%) receiving antiobesity medications required corticosteroids during the 12-month follow-up, a rate the authors noted was consistent with expected annual corticosteroid use in the general IBD population. Aksan et al^76^ found a statistically significant but clinically negligible reduction in the median number of steroid initiation instances over 5 years (median 2 vs 3 events, *P* < .0001, effect size r = 0.04). The systematic reviews by Thin and Teh^44^ and Maracle et al^62^ both noted that while large population-based registries reported reduced corticosteroid use among GLP-1RA users, smaller clinical cohorts showed no significant changes in corticosteroid prescription rates following GLP-1RA initiation. The evidence suggests that GLP-1RA use does not increase corticosteroid requirements in patients with IBD and may confer a modest protective effect in certain subpopulations, particularly those without obesity.

## Hospitalization

Evidence regarding hospitalization risk in patients with IBD using GLP-1RA demonstrates generally favorable or neutral outcomes across multiple studies. Gorelik et al reported that GLP-1RA use was associated with a significantly reduced risk of IBD-related hospitalization overall (aHR: 0.74, 95% CI: 0.61–0.91), with the effect being particularly strong in patients with UC (aHR: 0.63, 95% CI: 0.45–0.90) compared to patients with CD where the association did not reach statistical significance (aHR: 0.82, 95% CI: 0.65–1.05). The study also found significantly reduced composite outcome risk that included hospitalization in patients with BMI ≥30 (aHR: 0.61, 95% CI: 0.50–0.77).^72^ Villumsen et al^63^ similarly found a significantly decreased risk of IBD-related hospitalization with an adjusted incidence rate ratio of 0.73 (95% CI: 0.58–0.91). The meta-analysis by Bayoumi et al found no overall effect on hospitalization rates (HR: 0.91, 95% CI: 0.71–1.17, *P* = .47), with no significant differences in UC (HR: 0.88, 95% CI: 0.66–1.18) or CD (HR: 0.93, 95% CI: 0.78–1.10) subgroups. However, a sensitivity analysis limited to patients with BMI <30 showed a significant reduction (log HR: 0.79, 95% CI: 0.66–0.96, *P* = .01).^75^ Desai et al^64^ found no statistically significant differences in hospitalizations requiring IV steroids for either UC (aHR: 1.21, 95% CI: 0.92–1.59) or CD (aHR: 1.04, 95% CI: 0.80–1.34). Notably, Desai et al^71^ reported that semaglutide use in obese, nondiabetic patients with IBD was associated with a substantially decreased risk of any-cause hospitalization (aOR: 0.35, 95% CI: 0.19–0.67), though hospitalization requiring IV steroids showed no difference (aOR: 0.69, 95% CI: 0.29–1.61). Aksan et al found that GLP-1RA exposure was associated with significantly lower health-care utilization (χ2 = 136.52, df = 1, *P* < .0001).^76^ Multiple studies found no significant changes in IBD-related hospitalization rates following GLP-1RA initiation, including Levine et al^69^ (8.9% pre vs 7.6% post, *P* = .70), Anderson et al^73^ (10% pre vs 10% post, *P* = 1.00), and Weng et al^77^ (2.5% pre vs 0% post, *P* = .480). Pham et al reported that 2 of 36 IBD patients receiving antiobesity medications required hospitalization during the 12-month follow-up, a rate consistent with expected annual hospitalization frequency in the IBD population.^67^ These hospitalization findings were further supported by the systematic reviews by Thin and Teh^44^ and Maracle et al^62^ both of which noted that large population-based registries consistently reported reduced risks of hospitalization among GLP-1RA users, while smaller clinical cohorts showed stable hospitalization rates following GLP-1RA initiation. The cumulative evidence suggests that GLP-1RA use does not increase hospitalization risk and may provide protective benefits, particularly in certain patient subgroups.

## Adverse Events

### Common Gastrointestinal Adverse Events

Gastrointestinal adverse events are the most frequently reported side effects of GLP-1RA in patients with IBD, with Clarke et al^70^ reporting that 93% of adverse events were gastrointestinal in nature. Considerable variability in reported frequencies across studies likely reflects differences in agent selection, dosing protocols, patient populations, and study methodology (Table 5). Nausea was most prevalent (2.2%–31% across IBD studies; up to 51% in broader populations), followed by constipation (2%–25%), diarrhea (2%–12.5%), and vomiting (up to 8.3%).^65^^,^^66^^,^^68^, ^69^, ^70^^,^^74^^,^^77^ Other symptoms reported included abdominal cramping, decreased appetite, bloating, reflux, and indigestion.^66^^,^^69^^,^^70^ Pham et al^67^ reported gastrointestinal symptoms in 31.6% of IBD patients on GLP-1RA compared to 26.3% of matched non-IBD controls, with a higher discontinuation rate in the IBD group (11%) compared to controls (2.8%). Maracle et al noted in their systematic review that discontinuation rates across smaller cohorts ranged from 11% to 24%, primarily driven by gastrointestinal intolerance. Across the included studies, Maracle et al^44^ identified only 3 serious adverse events specifically attributed to GLP-1RA therapy: a cerebrovascular event, a case of severe diarrhea, and 1 case of pancreatitis, each leading to treatment discontinuation. These symptoms are typically dose-dependent, occurring most commonly during therapy initiation and dose escalation, and often overlap with baseline IBD symptoms, complicating clinical assessment.^65^

**Table 5:** Adverse Events Associated With GLP-1 Receptor Agonist Use in IBD

Adverse event	Study (year)	Incidence/rate	*P* value
Nausea	Ramos Belinchón et al (2024)^65^	13.3%	—
	St-Pierre et al (2024)^66^	31% (11/36)	—
	Pham et al (2024)^67^	8.3% (3/36)	—
Vomiting	St-Pierre et al (2024)^66^	8.3% (3/36)	—
	Pham et al (2024)^67^	5.6% (2/36)	—
Nausea/vomiting (combined)	Sehgal et al (2025)^74^	8%	—
	Clarke et al (2025)^70^	22%	—
Diarrhea	Ramos Belinchón et al (2024)^65^	12.5% (1 withdrawal)	—
	Pham et al (2024)^67^	8.3% (3/36)	—
	Sehgal et al (2025)^74^	2%	—
	Clarke et al (2025)^70^	6%	—
Constipation	St-Pierre et al (2024)^66^	25% (9/36)	—
	Pham et al (2024)^67^	5.6% (2/36)	—
	Sehgal et al (2025)^74^	2%	—
	Clarke et al (2025)^70^	11%	—
Ileus/obstruction	Desai et al (2024)^64^	UC: GLP-1 1.4% vs control 2.2%	*P* = .21
	Desai et al (2024)^64^	CD: GLP-1 3.1% vs control 3.6%	*P* = .56
	Bayoumy et al (2025)^75^	GLP-1: 1.0% vs control: 3.1%; OR 0.43	*P* = .18
	Nielsen et al (2025)^68^	GLP-1: 0.5% vs control: 3.1%; aHR 0.57	Significant
	Weng et al (2025)^77^	2.5% postinitiation vs5.0% preinitiation	*P* = .683
	Aksan et al (2025)^76^	GLP-1: 10.7% vs control: 12.3%	*P* < .0001
	Desai et al (2025, tirzepatide)^71^	<10 patients	—
Gastroparesis	Desai et al (2024)^64^	UC: GLP-1 1.6% vs control 1.09%	*P* = .31
	Desai et al (2024)^64^	CD: GLP-1 3.5% vs control 2.7%	*P* = .30
	Anderson et al (2025)^69^	0.57%	—
	Desai et al (2025, tirzepatide)^71^	<10 patients	—
Acute pancreatitis	Desai et al (2024)^64^	UC: GLP-1 1.19% vs control 1.11%	*P* = .87
	Desai et al (2024)^64^	CD: GLP-1 1.1% vs control 1.9%	*P* = .16
	Sehgal et al (2025)^74^	0.5%	—
	Anderson et al (2025)^69^	0.57%	—
	Desai et al (2025)^71^	GLP-1: 0% vs control: 0.13%	—
	Desai et al (2025, tirzepatide)^71^	<10 patients	—
Gallbladder/biliary events	Desai et al (2024)^64^	UC: GLP-1 4.8% vs control 3.3%; aOR 1.49	*P* = .11
	Desai et al (2024)^64^	CD: GLP-1 4.3% vs control 4.8%; aOR 0.89	*P* = .62
	Ramos Belinchón et al (2024)^65^	0%	—
	St-Pierre et al (2024)^66^	0%	—

aHR, adjusted hazard ratio; aOR, adjusted odds ratio; CD, Crohn's disease; GLP-1, glucagon-like peptide-1; OR, odds ratio; UC, ulcerative colitis.

### Ileus/Intestinal Obstruction and Gastroparesis

GLP-1RA delay gastric emptying, raising theoretical concerns about worsening bowel complications; however, the clinical evidence is reassuring.^60^ For ileus and obstruction, Nielsen et al^68^ found that GLP-1RA use was associated with a significantly lower risk of ileus or intestinal obstruction in the overall IBD population, with an aHR of 0.57 (95% CI: 0.36–0.88). This protective effect was particularly pronounced in patients with UC (aHR: 0.42, 95% CI: 0.21–0.86), while patients with CD showed a nonsignificant trend toward lower risk (aHR: 0.74, 95% CI: 0.42–1.30). Subsequent studies similarly demonstrated no increased risk or trends toward lower event rates across both UC and CD cohorts.^64^^,^^76^^,^^77^ The meta-analysis by Bayoumi et al^75^ found no clear difference in intestinal obstruction rates compared to controls (OR: 0.43, 95% CI: 0.13–1.49, *P* = .18).

Similarly, gastroparesis remains rare. Desai et al^64^ reported gastroparesis in 1.6% of UC and 3.5% of CD patients, with no statistically significant difference compared to other oral hypoglycemic agents (aOR: 1.50 and 1.30, respectively, both *P* > .05). Anderson et al^69^ reported gastroparesis in only 1 of 174 therapeutic trials of their IBD cohort, leading to medication discontinuation. Desai et al^78^ reported in their Tirzepatide study that fewer than 10 patients in the IBD cohort developed de novo gastroparesis or ileus, with event rates too low for formal statistical comparison. Overall, the evidence does not support increased risk of these motility complications with GLP-1RA use in IBD patients.

### Acute Pancreatitis

Although acute pancreatitis was an initial safety concern with GLP-1RA, evidence indicates comparable risk to other antidiabetic agents.^79^ Desai et al^64^ reported acute pancreatitis in 1.19% patients with UC and 1.1% of patients with CD with type 2 diabetes, with no statistically significant difference compared to other oral hypoglycemic agents (aOR: 1.07 and 0.58, respectively, both *P* > .05). Anderson et al^69^ and Sehgal et al^74^ each reported 1 patient with acute pancreatitis in their respective IBD cohorts leading to medication cessation. Notably, Desai et al^71^ reported no cases of acute pancreatitis in their obesity-focused IBD cohort during the follow-up period of 18 months. In a tirzepatide-focused study, Desai et al^78^ similarly reported that fewer than 10 patients in the IBD cohort developed de novo acute pancreatitis. Maracle et al^44^ identified 1 case of pancreatitis across the included studies that was specifically attributed to GLP-1RA therapy and led to treatment discontinuation. Overall, pancreatitis incidence with GLP-1RA is comparable to alternative glucose-lowering therapies and does not represent disproportionate risk in IBD populations.

### Gallbladder and Biliary Adverse Events

Gallbladder and biliary complications represent a recognized class-wide adverse effect of GLP-1RA, though evidence in IBD populations suggests no significantly elevated risk compared to control groups.^75^ Desai et al^64^ reported de novo gallbladder or biliary disease in 4.8% of patients with UC and 4.3% of patients with CD in their large propensity-matched database study, with no statistically significant difference compared to control groups (*P* = .11 and *P* = .62, respectively). Belinchón et al^65^ reported no cases of biliary complications during their 6-month follow-up period.

### Future Directions

The retrospective nature and significant heterogeneity of clinical outcomes in the existing studies limit the use of GLP-1RA as potential adjunctive therapies in the management of IBD. First, a clear mechanistic role of GLP-1RA use must be established to define how these agents suppress pro-inflammatory cytokines, modulate the microbiome, reduce macrophage and T-cell activation, and modulate the innate immune system in IBD. Future prospective and randomized controlled trials are needed with clearly defined IBD-specific outcomes and stratification by IBD subtype, GLP-1RA type, dose, BMI, and metabolic status. Future studies should also focus on the impact of GLP-1RA in CD stratified by disease location, severity, and phenotype. Furthermore, the use of GLP-1RA as combination therapies with biological agents or small molecules to induce remission or as add on therapies in patients who are partial responders also needs to be studied. Two placebo randomized controlled studies presently enrolling patients are examining the combination of mirikizumab and tirzepatide in patients with ulcerative colitis and Crohn's disease (NCT06937086 and NCT06937099). These studies are needed to position GLP-1RA as adjunct anti-inflammatory agents, especially in low-grade or metabolically driven inflammation seen in patients with obesity/metabolic syndrome. Finally, long-term safety and tolerability outcomes of GLP-1RA use in patients with IBD need to be studied.

## Conclusion

GLP-1RA is effective metabolic therapies for patients with IBD and coexisting obesity or type 2 diabetes mellitus. Observational studies demonstrate meaningful weight loss and improved glycemic control, without evidence of increased IBD exacerbation or need for escalation of therapy, including corticosteroids, hospitalization, advanced therapy initiation, or surgery. Importantly, available real-world evidence does not identify contraindications to GLP-1RA use in the general IBD population, and safety outcomes including ileus, intestinal obstruction, and acute pancreatitis are comparable to or no worse than control populations. Although mechanistic studies suggest potential anti-inflammatory effects through suppression of pro-inflammatory cytokines and enhancement of intestinal barrier function, clinical evidence for disease-modifying benefits remains limited by retrospective designs. Prospective randomized trials are needed to define long-term safety, efficacy, and the role of GLP-1RA as adjunctive therapy in IBD.
